# Raman and SEM analysis of a biocolonised hot spring travertine terrace in Svalbard, Norway

**DOI:** 10.1186/1467-4866-8-8

**Published:** 2007-08-15

**Authors:** Susana E Jorge-Villar, Liane G Benning, Howell GM Edwards, AMASE team

**Affiliations:** 1 Area de Geodinámica Interna, Facultad de Humanidades y Educación, C/Villadiego s/n, 09001 Burgos, Spain; 2 Earth and Biosphere Institute, School of Earth and Environment, University of Leeds, Leeds, LS2 9JT, UK; 3 Chemical and Forensic Sciences, University of Bradford, West Yorkshire BD7 1DP, UK

## Abstract

**Background:**

A profile across 8 layers from a fossil travertine terrace from a low temperature geothermal spring located in Svalbard, Norway has been studied using both Raman spectroscopy and SEM (Scanning Electron Microscopy) techniques to identify minerals and organic life signals.

**Results:**

Calcite, anatase, quartz, haematite, magnetite and graphite as well as scytonemin, three different carotenoids, chlorophyll and a chlorophyll-like compound were identified as geo- and biosignatures respectively, using 785 and/or 514 nm Raman laser excitation wavelengths. No morphological biosignatures representing remnant microbial signals were detected by high-resolution imaging, although spectral analyses indicated the presence of organics. In contrast, in all layers, Raman spectra identified a series of different organic pigments indicating little to no degradation or change of the organic signatures and thus indicating the preservation of fossil biomarker compounds throughout the life time of the springs despite the lack of remnant morphological indicators.

**Conclusion:**

With a view towards planetary exploration we discuss the implications of the differences in Raman band intensities observed when spectra were collected with the different laser excitations. We show that these differences, as well as the different detection capability of the 785 and 514 nm laser, could lead to ambiguous compound identification. We show that the identification of bio and geosignatures, as well as fossil organic pigments, using Raman spectroscopy is possible. These results are relevant since both lasers have been considered for miniaturized Raman spectrometers for planetary exploration.

## Background

There is currently much interest in the detection and characterization of biosignatures in geological environments for planetary exploration; instrumental suites for life detection are being considered for several extreme terrestrial environments, such as Arctic and Antarctic cold deserts, hypersaline lakes, and for remote robotic surface and subsurface exploration on Mars [1-6].

A combination of analytical techniques is extremely valuable for obtaining complementary information about complex biogeological systems [4,7-11]. In this respect, Raman spectroscopic instrumentation is being evaluated for the adoption of miniaturized versions as part of life detection suites on future Mars missions [12-15]. An important part of this scenario is the acquisition of molecular spectroscopic data signatures from key biomolecules produced as a consequence of extremophile survival in stressed environments; a range of terrestrial Mars analogues have been examined including endoliths, cyanobacteria from meteorite impact craters, stromatolites or volcanic and saline rocks [16-21].

The acquisition of Raman spectral data in conjunction with a surface morphological and an elemental analysis from scanning electron microscopy has a special advantage in that the extraction of biological material from the geological matrix is not necessary and no sample handling or preparation steps are needed; hence, it is possible to obtain direct data on the interactions between organic and inorganic components in a biogeological system. From such data the survival and preservation potential of extremophile biosignatures can be accessed.

From these studies, key Raman biosignatures of organic chemicals produced by organisms in rocks have been established and will be applied here to the study of the remnant biosignatures in a travertine terrace in Svalbard (Norway) using samples collected during the 2004 AMASE (Arctic Mars Analogue Svalbard Expedition) expedition. The combination of scanning electron microscopy and Raman microspectroscopy applied to this biogeological system will facilitate the characterization of morphological and biomolecular data for these Mars analogues for the first time.

### Specimens

Samples were collected in the Bockfjorden area of Spitzbergen (Svalbard archipelago, Norway) from the fossil parts of the northernmost warm springs on land, the Troll springs, which are located at N 79 °23' and E 13 °26'. The springs are linked to the Bockfjorden Volcanic Centre, which is located along a major N-S trending fault-zone separating Devonian red sandstones from Proterozoic basement rocks [22].

**Figure 1:**
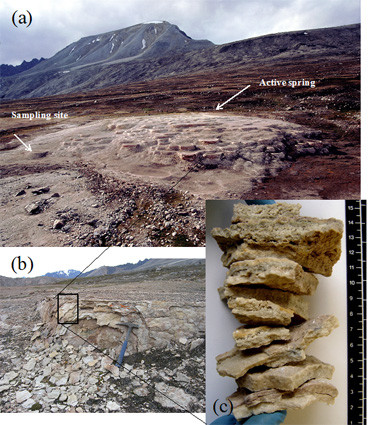
Image of the hotspring, terrace and profile of the analysed layers (a) Overview of the Troll Springs. Indicated are the position of the active part of the spring and the location of the sampling site in the oldest, fossil part of the springs. (b) detailed image of sampling site. (c) Profile of the 8 layers investigated in this study, top young, bottom, old.

The Troll springs are active today only at the topmost end, while the largest part of the terraces created during the lifetime of the springs are now considered fossil remnants of active terraces (Figure 1a). The Troll spring terraces span about 100–120 m in the north south direction and, besides the active pool, they contain many dry, fossil pools of sizes between 2–10 meters. These dry pools often have lips of up to 50 cm in width and draped overflow edges that can reach several meters in height. The full vertical dimension of the terraces, measured from the today active site and including all the fossil parts varies between 30–50m.

The samples studied here were collected from the draped overhang of an old pool located at the south-easternmost end of the system. This part is considered to be one of the oldest, fossil parts of the Troll terraces (Figgure 1b). A traverse consisting of 8 layers with the external, youngest one (layer 1) being underlaid by 7 progressively older layers, was collected and each layer analysed. The thickness of the individual layers varied between 0.7 and 1.8 cm and they exhibited a range of colorations from pure or dirty white to pinkish to slightly greenish or yellow and brown (Figure 1c). Observation of the surrounding rocks showed no indication of any recent water flow except water derived from precipitation. The general appearance of the layers were not indicative of classical cryptoendolithic strata, nor were any clear endolithic or casmolithic signatures observed. However, the slight colouring of certain strata indicated possible endolithic colonization and/or this coloration could be derived from variation in elemental content, i.e. brown, yellow indicating higher iron content or pink indicating higher Mn content [23]. The collected samples were analysed with no further sample preparation and in a consecutive mode with Raman analyses (no sample preparation, except breaking under sterile conditions, flow cabinet) being followed by high-resolution microscopy and elemental analyses (coating and in vacuum). This sequentially analyses protocol was followed such that the same samples and spots could be studied. Note that samples were also collected for normal microbiological preservation methods (i.e., fixed in the field in glutaraldehyde), however, the thrust of the work presented in this paper was carried out on the untreated samples. The preserved samples were solely used for high-resolution microscopy and elemental analyses to cross-check the preservation or degradation of possible morphological biosignatures during sample transport and storage. Because the organic signatures from glutaraldehyde compromise the Raman analyses naturally only the untreated samples were analysed by Raman.

### Raman spectroscopy

Spectra were recorded using a Renishaw *InVia*spectrometer with a Leica DMLM microscope and objectives with × 50 and × 20 magnifications. Two laser excitation wavelengths were used for the analyses (785 and 514 nm) in order to determine and quantify the differences between Raman signatures and to derive the best protocol for analyses of extant biosignatures. For each measurement, between 20 and 70 spectral accumulations at 10 seconds exposure time were recorded to improve signal-to-noise ratios. In addition, in order to avoid sample damage, low laser powers between 0.5 and 2.5 mW at source were used. Each layer was analysed with no further preparation being undertaken.

### Scanning electron microscopy

Following the Raman analyses, the morphology and distribution of mineral phases and organic matter in the samples were imaged using a LEO 1530 Field-Emission Gun Scanning Electron Microscope (FEG-SEM). Qualitative elemental analyses were also carried out using an energy dispersive spectrometer (EDS) system that was attached to the FEG-SEM. With no further handling or preparation the samples were placed on an aluminium SEM stub and coated with a 3 nm platinum layer. Samples were inserted into the vacuum and imaging was done at an accelerating voltage of 3 kV and a working distance of 3 mm while EDS spot; analyses were carried out at 15 keV and a working distance of 8 mm. The samples that were preserved in the field in glutaraldehyde (in PO_4_buffer at pH 7) were sequential dehydrated in an ethanol series (10, 30, 50, 70, 90 and 100 %) and imaged as described above. Critical point drying of the so prepared samples was unnecessary.

## Results and discussion

### Mineralogy

**Figure 2:**
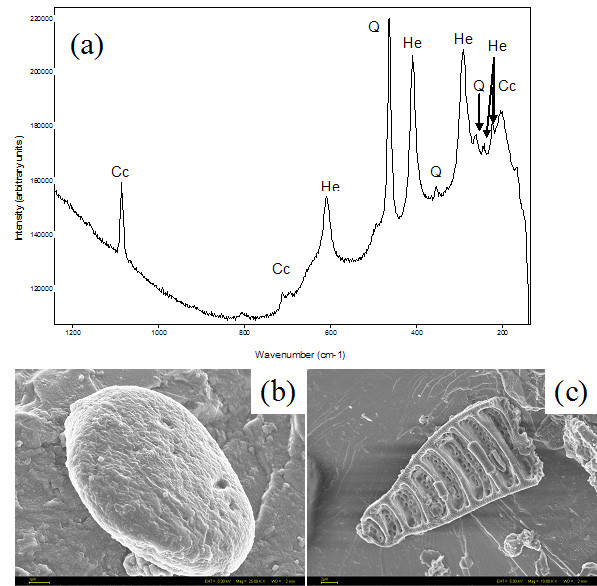
Representative Raman spectrum (a) of quartz (Q), calcite (Cc) and hematite (He) and FEG-SEM images of a dust particle(b) and a diatom (c) recorded at 785 nm laser wavelength. When analyzed using EDS both (c and d) showed Si and O peaks in the spectra. Scales 2 and 1 μm respectively.

SEM-EDS shows that the dominant mineral in all layers was a calcium carbonate, which was identified as calcite based on the crystal morphology. Using Raman spectroscopy the calcium carbonate was confirmed as calcite, with bands at 1086, 712, 281 and 157 cm^-1^. Several accessory minerals were also confirmed by Raman spectroscopic analyses, such as haematite, quartz, magnetite, graphite and anatase. Hematite was confirmed via the signatures at 224, 245, 296, 410 and 613 cm^-1^, and it was present particularly in the yellowish layers. Quartz (Figure 2a), was identified based on bands at 204, 263, 353, 406, 508 and 1064 cm^-1^, and its source was deemed to have been wind-borne dust (Figure 2b). Magnetite was detected in some spectra via a band at 664 cm^-1^. SEM images also revealed the presence of various diatoms (Figure 2c) but a Raman signature for amorphous silica (opal A, broad bands expected at ~1100 and 800 cm^-1^) was not identified. This is most probably due to the poorly ordered nature of the silica in opal A and its interference with other more crystalline Raman mineral signatures.

**Figure 3:**
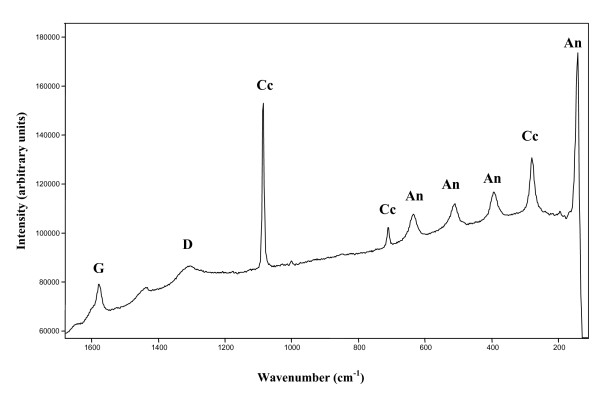
Raman spectrum of three minerals unambiguously identified by their Raman bands. Representative Raman spectrum of graphite (D 1308 cm^-1^and G 1572 cm^-1^); anastase (An), with bands at 637, 512, 394, 196 and 145 cm^-1^and calcite (Cc), 1086, 712, 281 and 157 cm^-1^recorded at 785 nm laser wavelength. The broad band around 1400 cm^-1^could not be unambiguously assigned.

Graphite was present in several layers and it was characterised by a strong and sharp G Raman band at 1572 cm^-1^and a broader and weaker D band at 1308 cm^-1^. The presence of graphite in the Troll springs is most probably related with aerosol transport from the antracite outcrops in the southern part of the island. Lastly, anatase, with bands at 637, 512, 394, 196 and 145 cm^-1^, has been identified in several layers (Figure 3).

### Biosignatures

Layers were analysed using the 785 and 514 nm laser excitation wavelengths and the organic signatures detected were assigned to scytonemin, carotene, chlorophyll and an unidentified organic compound (a porphyrin with a structure close to chlorophyll). It is important to highlight here that the Raman spectrum of scytonemin showed significant variations in the relative intensity of the bands when recorded using the 785 or 514 nm laser excitation and this could lead to ambiguous pigment identification.

It is also noteworthy to point out the differences in organic component admixtures observed when using the different laser excitations on the same analyses spot. Several layers analysed with the 785 mn near infrared laser, revealed specific admixtures of pigments a) scytonemin and carotene, b) scytonemin, carotene and chlorophyll, c) carotene and chlorophyll. Conversely, at a 514 nm laser excitation wavelength the admixture of pigments detected on the same location was a) carotene and scytonemin and b) carotene only. In this study, carotene was never found on its own when using the 785 nm laser excitation but it always appeared with chlorophyll or scytonemin or both; however, using the 514 nm laser wavelength chlorophyll has not been identified in any of the spectra.

Raman signatures also give information about the degradation of organic compounds or the crystalinity of materials. For organic compound, the presence of sharp bands indicates, in general, that the molecules are not degraded; usually upon degradation the organic bonds become weaker, and the Raman bands appear broader; such a broadening indicates that the organic compounds have to some degree degraded. Similarly, for minerals broader Raman bands indicate lower levels of crystallinity.

### Scytonemin

**Figure 4:**
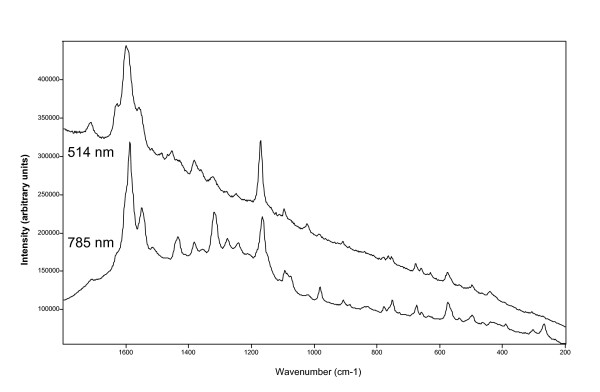
Representative Raman spectrum of scytonemin recorded at two different laser excitation wavelengths. The different relative intensities of the bands makes the compound identification difficult. For a more detailed comparison see Table 1.

The spectra of scytonemin exhibit numerous Raman bands regardless if collected at 514 or 785 nm laser wavelength (Figure 4, Table 1). The most significant difference was observed in the relative intensities for specific bands when collected at the two laser excitations which was expressed in changes the overall appearance of the scytonemin spectra; this applied for both the corroborative Raman signatures as well as minor bands (Figure 4). Such differences could make a definitive compound identification difficult and this is specifically true if admixtures with other organics or minerals are present and band overlaps or interferences are observed.

**Table 1:** Scytonemin Raman bands collected using 514 and 785 nm laser wavelength excitations.

Scytonemin (514)	Scytonemin (785)
1630_s_	1627_vw_
	1605_sh_
1599_vs_	1590_vs_
1591_vs,sh_	
1557_s_	1554_s_
1436_m_	1436_s_
1382_m_	1384_m-w_
1363_m-w_	
1323_m-w_	1320_s_
1282_m-w_	
	1278_m-w_
1250_w_	
	1242_m-w_
1171_vs_	1171_vs_
	1090_w_
1097_m_	
1023_m_	1023_vw_
985_vw_	982_m-w_
955_w_	
909_m-w_	908_w_
	886_w_
779_vw_	
	779_w_
764_vw_	
752_vw_	752_m-w_
677_m_	676_m_
660_m-w_	659_w_
640_vw-sh_	
629_w_	
576_m_	574_m_
538_vw_	
496_m-w_	495_m_
428_w-sh_	428_vvw-sh_
441_m_	441_vw_
396_vw_	392_w_
306_vw_	

For example (Table 1), the band at 1627 cm^-1^was weak when the spectrum was collected at 785 nm, but at 514 nm the relative intensity increased and, moreover, the band shifted and appeared as a triplet at 1630, 1599 and 1557 cm^-1^. In addition, at 785 nm the strongest band was observed at 1590 cm^-1^while with the 514 nm excitation this band shifted to 1599 cm^-1^with a very strong shoulder at 1591 cm^-1^; lastly, in the spectrum collected using 785 nm laser wavelength, the strong band at 1554 cm^-1^appeared at 514 nm as part of a triplet with medium intensity.

Another corroborative band for scytonemin can be found at 1320 cm^-1^(medium intensity) but when the spectrum was collected with the 514 nm wavelength excitation the relative intensity of this band decreased significantly and the less significant signature at 1382 cm^-1^, became stronger.

The spectral differences observed in the 1000–350 cm^-1^region are not as significant as those in the 1800–1000 cm^-1^. Usually these bands are not used as corroborative signatures; however, distinct differences have been observed between the spectra collected using the two laser excitations (Figure 4, Table 1). For example, in the spectrum collected with 785 nm the band at 982 cm^-1^showed a medium-weak intensity while at 515 nm this band appeared only as a very weak band. Some further differences were noted in the triplet at 779–764–752 cm^-1^(514 nm laser), which became a band at 752 cm^-1^with a very weak shoulder at 764 cm^-1^and a weak signature at 779 cm^-1^when the Raman spectrum was collected with the 785 nm laser excitation. Lastly, the weak band at 629 cm^-1^with a shoulder at 640 cm^-1^and the signature at 441 cm^-1^with a shoulder at 428 cm^-1^(using a 514 nm laser excitation wavelength), appeared just as very weak intensities in the spectrum collected using the 785 nm laser excitation.

#### Significative band shifts in scytonemin spectra

**Figure 5:**
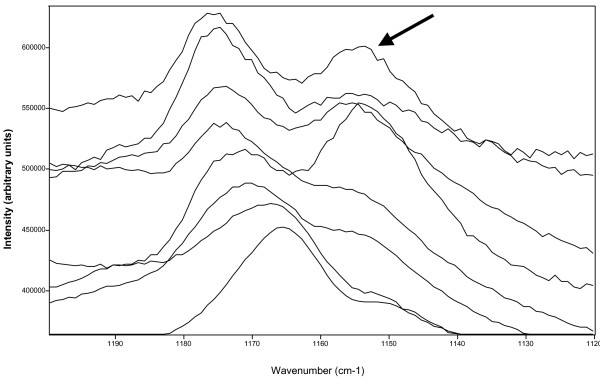
Stackplots of 8 scytonemin spectra recorded at 785 nm. The arrow shows the carotene band at around 1515 cm^-1^, which is not related with the shift in scytonemin bands from 1172 cm^-1^to 1165 cm^-1^(marked by triangles), since no carotene is observed in the lower spectrum.

When the spectrum of scytonemin was collected using the 785 nm laser wavelength there were also significant shifts observed in the second main corroborative scytonemin band; according to the literature [24], the signature for this band should be centred at 1172 cm^-1^but in this study we have observed that this band shifted from 1176 to 1165 cm^-1^(Table 2 and Figure 5). It needs to be noted that this effect was not related to the presence of carotene (which exhibited a strong band centred about 1155 cm^-1^). In Figure 5, stack plots of eight scytonemin spectra recorded in the region between 1200–1120 cm^-1^are shown and only some of the spectra in this figure display the carotene signature (arrow in Figure 5). However, the spectrum with the lowest wavenumber for the prime scytonemin Raman band at xxx cm^-1^(e.g., lowermost spectrum) does only reveal a small shoulder at ~1150 cm-1 and this could be indicating the presence of carotene. The shift observed for this significant scytonemin Raman signature (lowermost to uppermost spectra from 1165 cm^-1^to 1176 cm^-1^) can thus not be attributed the carotene band overlap.

**Table 2:** Scytonemin Raman band shifts collected in the eight studied layers at 785 nm laser excitation wavelength (SCY1 to SCY8 represent spectra in the eight studies progressively older layers). There is no relationship between the position of the individual scytonemin bands and the depth profile in the different layers and two different assignments may be present in the same layer or the same assignment may be valid in different layers.

SCY-1	SCY-2	SCY-3	SCY-4	SCY-5	SCY-6	SCY-7	SCY-8
				1711_w_	1711_w_	1711_w_	1711_w_
	1630_vw_			1630_vw_			
					1629_w_	1629_w_	
1627_vw_			1627_vw_				
							1624_vw_
1605_sh_	1605_sh_	1605_sh_	1605_sh_	1605_sh_	1605_sh_		1605_sh_
						1603_sh_	
1592_vs_	1592_vs_						
		1591_vs_			1591_vs_		
			1590_vs_	1590_vs_		1590_vs_	
							1589_vs_
1555_s_	1555_s_						
				1554_s_	1554_s_		
		1552_s_					
			1551_s_			1551_s_	
							1549_s_
1436_s_	1436_s_		1436_s_	1436_s_	1436_s_	1436_s_	
		1435_s_					1435_s_
1385_m_		1385_m_	1385_m_				
	1384_m_			1384_m_	1384_m_		
						1383_m_	1383_m_
			1325_s_				
		1324_s_					
					1322_s_	1322_s_	
1320_s_	1320_s_			1320_s_			1320_s_
					1280_m-w_	1280_m-w_	
				1278_m-w_			
							1277_m-w_
						1249_w_	
					1245_m-w_		
							1243_m-w_
				1242_m-w_			
**1176**_vs_							
	**1175**_vs_						
		**1174**_vs_					
			**1173**_vs_				
				**1171**_vs_			
					**1170**_vs_		
						**1167**_vs_	
							**1165**_vs_
						1095_w_	1095_m_
				1090_w_	1090_m_		
						1026_vw_	
1024_vw_							1024_w_
				1023_vw_	1023_w_		
			984_w_				
983_w_	983_m_	983_m_				983_m_	
				982_m_	982_m_		982_m_
			910_vw_				
909_vw_	909_vw_	909_w_					
				908_w_	908_w_		908_w_
						907_w_	
			890_vw_				
	889_vw_	889_w_					
887_vw_					887_w_	887_vw_	887_w_
				886_w_			
						779_w_	
		778_w_		778_vw_	778_w_		
							777_w_
754_w_			754_m_				
	753_w_						
		752_m_		752_m_	752_m_	752_m_	752_m_
	680_vw_						
677_vw_			677_m_				
		676_m_		676_m_	676_m_		
						675_m_	675_m_
	662_w_						
		660_w_					
				659_w_	659_w_		
						658_vw_	658_w_
					637_vw_	637_vw_	
	578_w_						
			576_m_				
575_w_		575_m_					
				574_m_	574_m_	574_m_	574_m_
	499_m-w_						
497_vw_		497_m-w_				497_w_	497_m-w_
					496_m-w_		
			495_m-w_	495_m-w_			
					437_w_		
				392_w_	392_w_		
							390_w_

Although there is a minor shift in other strong scytonemin observed from 1555 to 1549 cm^-1^(Table 2), the tendency to shift to a lower wavenumber was not as clear as as the band at 1172 cm^-1^and it is clearly not related with it, neither with the very strong signature between 1592 to 1589 cm^-1^.

### Chlorophyll and chlorophyll-like pigment

In several layers chlorophyll was identified using the 785 nm laser excitation by its Raman bands at 1625, 1580, 1436, 1372, 1323, 1285, 987, 916, 744 and 515 cm^-1^. Note that, as mentioned above, chlorophyll was never identified using the green laser at 514 nm excitation wavelength.

**Figure 6:**
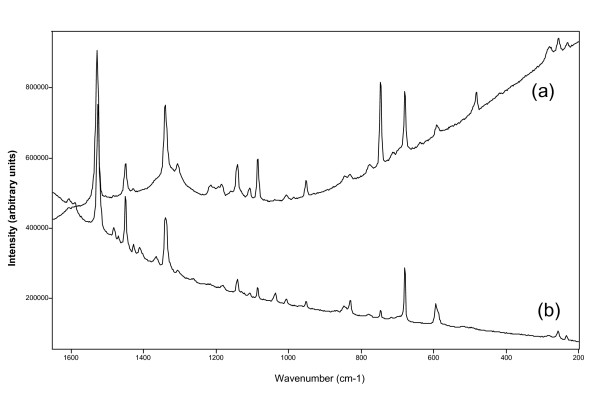
Spectra recorded of the chlorophyll-like compound using the (a) 785 nm and (b) 514 nm laser wavelengths. Important differences in relative intensities of the main corroborative Raman bands are shown. For more detailed information see Table 3 and text.

Another organic compound, with a similar structure to chlorophyll (porphyrin), showed signatures at 1592, 1530, 1485, 1451, 1428, 1411, 1307, 1216, 1194, 1144, 1108, 953, 847, 832, 779, 746, 679, 640, 594 and 483 cm^-1^and it was detected with both lasers in several layers. However, again differences in the Raman spectra collected with the two lasers (Figure 6, Table 3) were observed. The relative intensity of the corroborative signatures at 1339 cm^-1^with a second band at 1307 cm^-1^and the signature at 747 cm^-1^was stronger in the spectrum collected with the 785 nm laser excitation in comparison with that collected using the 514 nm laser excitation. In addition, the medium intensity signature at 483 cm^-1^, observed using the 785 nm excitation, was not detected when the Raman spectrum was collected with the 514 nm laser wavelength.

**Table 3:** Raman bands of the clorophyll-like compound recorded with the 514 or 785 nm laser wavelengths. Note that the relative band intensities change significantly with the wavelength.

514 nm laser excitation	785 nm laser excitation
1592_vw_	1592_vw_
1530_s_	1530_s_
1485_m-w_	1485_vw_
1451_s_	1451_m_
1428_w_	1429_w_
1411_w_	
1339_s_	1341_vs_
1307_w_	1307_m_
	1219_w_
1194_w_	1194_m_
	1144_vw_
1108_vw_	1108_m-w_
1037_m-w_	
953_m-w_	952_m_
847_vw_	847_wv_
831_m_	831_w_
	779_w_
746_m-w_	746_vs_
680_s_	679_s_
	640_vw_
594_m_	591_m-w_
	483_m_
257_m-w_	257_m-w_
235_w_	235_w_
175_w_	175_w_

### Carotenoids

**Figure 7:**
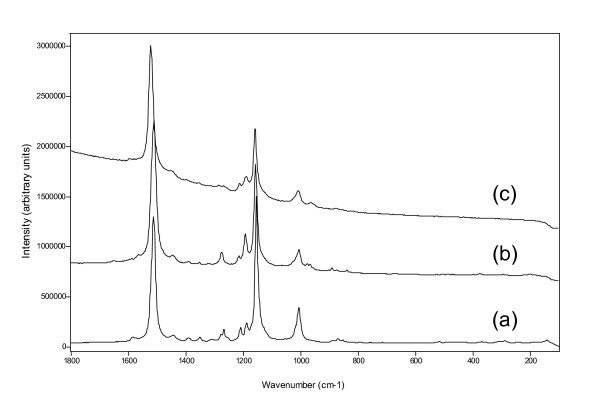
Representative Raman spectra of carotenoids found in the layers. (a) beta-carotene, (b) astaxanthin, (c) zeaxanthin/lutein, recorded at 514 nm laser wavelength. The carotene detection is easier with the 514 nm laser wavelength because of the resonance effect.

Several different carotenoids have been identified by their Raman signatures (Figure 7). The mayor bands at 1508, 1154 and 1005 cm^-1^represent a carotene with a long chain structure and this could be assigned to astaxanthin. Another carotene exhibited bands at 1515, 1155 and 1003 and these are characteristic for beta-carotene while a third carotene, with the shortest chain length, showed Raman bands at 1522, 1157 and 1007 cm^-1^and these are assignable to zeaxanthin or lutein.

### Morphological biosignatures

The microscopic imaging and spectral analyses of all layers showed that the main mineral in all layers was calcite. In addition, the presence of particles of dust, pollen grains and diatoms were discovered in some of the layers. EDS analyses of the mineral phases naturally showed C, O and Ca as peaks for the calcite substrate. In addition, sometimes Fe and Mn peaks were observed and these could be linked to the yellow-brown or white-pink layers.

**Figure 8:**
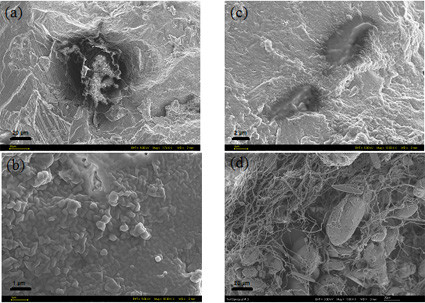
Representative FEG-SEM images of organic structures observed in the fossil terrace layers (a-c) compared to a fresh sinter rim sample from the active pool rim(d): (a) large organic matter cluster that showed no distinguishing morphological microbial features even at higher resolution (b). Only one structure could be interpreted as possible microbial remnants (c). All these structures when analyzed under EDS exhibited a stronger C peak than the calcite substrate; in addition these organic clusters were easily degraded under the electron beam (beam damage). For comparison (d) shows filamentous microbes in a sinter piece from the active part of the main terrace. Scales (a) and (d) 20 μm; (b) 1 μm; (c) 2 μm.

However, the detailed study of all layers failed to locate any indication of clear and distinguishing morphological features that resemble bacteria, algae or fungi. The only organic looking structures found in some layers were fuzzy or diffuse structures and blebs of organic matter. (Figure 8a and 8b). These features when analysed under the electron beam degraded fast, yet the EDS analyses showed a stronger C signal and sometimes a P signal when compared with the pure calcite substrate. Only in one case was one feature found that could be interpreted as a possible remnant of bacteria (Figure 8c).

The features in Figure 8a–c were initially discovered during the Raman analyses and they clearly showed Raman signatures for bona fide scytonemin and carotenoids. The same location was subsequently imaged and spectrally analyses using the FEG-SEM. Although the images clearly showed that the organic matter discovered had no morphological resemblance to microbes, it is also clear form the Raman spectra that this organic matter must have been microbial in origin. The Raman signatures on the globule shown in Figure 8a clearly demonstrated the presence and thus persistence and preservation of microbially derived scytonemin and carotenoids. As a test, we imaged and analysed a fresh sample from the edge of the active pool at the top of the terrace (see arrow in Figure 1) and clearly demonstrated that during the growth of the terraces abundant and diverse microbial communities as well as diatoms are present (Figure 8d) and that the biosignatures we documented in the fossil layers can only be derived from microbes that were present during growth of the sinter but that have been totally altered and degraded during aging.

### Terrace evolution and preservation of organics

It is clear from the general aspect of the fossil part of the Troll springs that at some time the extent of the active part must have been much bigger than it is observed today. In addition, the multitude of large dry pools and their sizeable aprons, lips and draped sides (Figure 1a, b) indicate higher flow rates in the past when compared with the gentle sloped terraces that form in the present. Although it is not possible to provide an age of the various layers or of the terraces themselves, from calcite growth rate studies carried out in the active parts of the springs during a previous AMASE expedition [22,23] it is possible to roughly approximate an age for each individual layer and extrapolate to a possible age of the sequence studied here.

Hammer *et al *[22] (2006) estimated a maximum growth rate in the active springs of ~2 mm/year (minimum 0.1 mm/year). It has to be pointed out that this was done for slow growing sinters at temperatures between 12 and 23°C and under the assumption of continuous growth (over a one year period) and not taking into account seasonal variations, compaction due to later dehydration or aging. In addition, these variations in growth rates were strongly dependent and calcium carbonate saturation, which were in turn linked to water temperature (12–23°C) and depth (1–10 cm) as well as flow and evaporation rate. Recently, Jamtveit *et al *[23] (2006) used spatially resolved isotopic measurements along a 5 cm piece of travertine collected from the middle part of the fossil terrace (see Figure 1 in this paper and Figure 2 in Jamtveit et al 2006). Their results showed that growth rates could be as high as 5.5 mm/year but they also tracked temperatures in the spring and surrounding air for 12 months and demonstrated that for about 6 months per year the springs are frozen. This will alter the overall growth rates because the cold temperatures will allow for no or only little growth to occur in the winter months and thus each year only a maximum of ~2.75 mm would be added to each layer.

Lastly, it is also important to note that certain parts of the terraces can become dry and others can be reactivated after prolonged dry periods (based on consecutive visits to the springs during 2003 to 2006 and historical records). Such drying out and subsequent reactivation of growth can lead to the formation of the observed gaps between layers (Figure 1b and 1c). When flow directions change and thus, for example an overflow dries this will lead to compaction and the formation of a harder crust. Once the same overflow becomes active again the next layer can grow. The observed gaps may also be affected by the past-presence of biological materials (i.e., exopolymer-rich biofilms) that would have dried up once water flow ceased. From the analyses of the modern equivalents of these fossil terraces, filamentous microbial colonies were observed in the fresh sinter pool edges (Fig. 8d) yet the observed gaps (Fig. 1b) can not be solely explained by biological processes and thus the drying and reactivation of sinter growth and compaction in the layers with time is the more likely cause for the observed layered structure of the terraces.

Based on these observations we determined a minimum and maximum possible growth rate for each layer in our traverse. The 8 layers studied varied in thickness between 7 and 18 mm and were distinguished by relatively large void spaces that indicated dry periods. If we take the previously determined growth rates (0.1 – 2.75 mm/year) and assuming that each layer grew in one continuous event, we can determine that the layers in our traverse could have taken between a minimum of 2.5 and a maximum of 180 years to grow.

However, it needs to be pointed out that the voids between the 8 layers in the traverse (Figure 1b and 1c), as well as the differences in density and porosity of each layer (Figure 1c) are all strong indication of prolonged dry periods between layer deposition. This naturally makes an estimate of the age of the whole sequence difficult. Based on our observations between 2003 and 2006 as well as historical evidence (i.e., Hoel and Holtedhal, 1911 and Banks et al 1998) we can infer that such dry periods may last between 2 and 20 years. Using a median growth rates for each layer of ~50 years we can estimate a ballpark upper maximum time for the growth of the studied 8-layer sequence of ~400 to 550 years. Such an estimate is also supported by the fact that the last glaciation in the area was ~8000 years ago and that the springs must have been formed since that time, else they would have been eroded. Although no exact date for the ages of the fossil terraces is available the sequence studied here was collected from the oldest part of the terrace (Fig. 1a) and thus it is highly probably that there may have been no active flow for several hundreds of years. In addition, the lack of any evidence of endolithic or casmolithic signatures, support the fact that little to no biocolonisation of these sinters occurs since deposition. Note however, that the error in these age estimate are invariably large but the purpose of this calculation was solely to provide a possible upper time limit for the formation of the studied sinters. Combined with our Raman and SEM results that showed that specific microbially derived organic signatures have been preserved virtually unchanged in these old fossil terrace layers despite there being no clear indication of modern activity or clear remnant morphological biosignatures.

## Conclusion

All geological layers show evidence of organic colonization with the presence of scytonemin, carotene, chlorophyll and/or chlorophyll-like compound, showing that a continuous colonization has occurred during the terrace formation. This confirms the observations of Hammer *et al*[22] (2006), who showed clear involvement of microbial communities in the build up of the new active terraces (see also Figure 8d). Here we showed, that Raman microspectroscopy in combination with SEM imaging and elemental analyses can be used to detect biological signatures that have been preserved for long periods of time even if remnant morphological evidence is missing. In addition, this can be done without chemical extraction or sample handling. Our results showed that, in all layers, some of the colonizing organisms have produced scytonemin during their lifetime and each layer must have contained at least one of the scytonemin producing organisms. In addition, another type of microorganism which produced a chlorophyll-like compound was only found in one layer but it was detected using both laser wavelengths.

Although scytonemin has been detected with both laser excitation wavelengths the variations in relative band intensity strongly alters the appearance of the Raman spectra and this could make it difficult for unambiguous compound identification. The same problem was observed with the chlorophyll-like compound where some corroborative bands were absent when the spectrum was collected using 514 nm laser excitation.

It is relevant that although no morphological bacterial remains were found using SEM, the Raman spectra showed sharp bands indicating little to no pigment alteration; this indicates that although the original microbial community could not be observed via its morphological biosignatures and only organic matter has been preserved, the pigments themselves have been fully conserved. This is specifically interesting as these pigments have survived in an environment with alternating dry and wet and cold and temperate periods over a long period of time. These results are particularly significant for planetary exploration since we showed that biosignatures could remain unalterated and thus remain preserved for decades to centuries. In addition, we show that their detection and quantification is possible by Raman spectroscopy, without any sample manipulation (except those derived of the exposition of a fresh surface for analysis by breaking the rock).

As Raman spectroscopy has been proposed as a part of a suite of analytical techniques for planetary exploration, particularly for Mars surface and subsurface future NASA and ESA missions [25-27] and both laser wavelengths tested in this study (and others like 532, 633 or 1065 nm) have been considered for miniaturised Raman spectrometers, the selection of the most adequate and most specific wavelength for organic detection is of prime significance. As we highlight in this study, the laser wavelength affects the relative intensity of the bands, making difficult the unambiguously identification of biosignatures. However, it is important to point out that the capability of detection of organic pigments could be compromised if the wrong laser wavelength is used.

The requirements for design of a miniaturised Raman spectrometer for planetary exploration should be related not only to its weight, robustness or other technical characteristics but also with the scientific aims of the research goals. In this respect, laser wavelength as well as spectral resolution or laser power are of vital importance for the success of the search for life missions to extraterrestrial planets. Naturally only when combined with microscopy, elemental or even isotopic analyses could extinct or extant biosignatures be unambiguously distinguished in fossil terrestrial and non- terrestrial environments.

## Authors' contributions

SEJV and LGB together collected the samples during AMASE 2004, and carried out the SEM analyses and imaging. SEJV and HGME carried out the Raman analyses. SEJV, LGB and HGME have all equally contributed to writing the manuscript. The AMASE team has helped during the expedition with logistics and support.

All authors have read and approved the final manuscript.
